# The effect of statins on National Institutes of Health Stroke Scale (NiHSS) in Ischemic stroke

**Published:** 2015

**Authors:** E Hamzei, A Alavi, M Khorami Saber, M Azad

**Affiliations:** * Clinical Research Development Center of Shahid Mohammadi Hospital, Hormozgan University of Medical Sciences, Bandar Abbas, Iran; ** Student Research Committee, Hormozgan University of Medical Sciences, Bandar Abbas, Iran

**Keywords:** statins, stroke, ischemic

## Abstract

**Introduction.** Stroke is a type of nervous focal disorder that occurs suddenly because of acute vascular events. 113-149 out of 100000 persons (0.113-0.149%) face with stroke in Iran annually. This causes treatment costs and many disabilities. Recently, studies indicated that statins, as a class of drugs, could decrease the chance of stroke recurrence and probably disabilities after stroke onset. This paper presents the effects of previous statins consumption on NIHSS in patients hospitalized in Shahid Mohammadi Hospital of Bandar Abbas in 2014-5.

**Materials and methods.** Based on the World Health Organization categorization, 140 patients suffered from stroke, experienced stroke once or more, and were older than 20 years old. They were studied with a simple convenience sampling in this descriptive study during 2014-5. The exclusion criteria were non-neurological vision disorders, ICH, and unwillingness of the patients to participate in the research. The patients were classified into two teams, one taking statin before the stroke onset (group A) and other taking no statins before stroke onset (group B). Patients were initially examined with the help of NIHSS rate measurement (using NIHSS rating standard questionnaire), type of stroke (ischemic or hemorrhagic), and other demographic data. Then the data were inserted into SPSS 22 and descriptive statistical tests (median, mean and standard deviation), Spearman and Mann-Whitney tests, were used due to the abnormal distribution of the data.

**Results.** 140 patients suffered from stroke (95 men (67.85%) and 45 women (32.14%)) with an average age of 67.9 years old, being studied in two groups (A and B). A direct correlation was seen between age and NIHSS (sig = 0.057) but it was not notable (P > 0.05). There was no clear relationship among sex, number of prior stroke onsets, type of stroke, lipid profile (TG, Total Cholesterol, LDL, HDL), blood sugar, underlying disease (diabetes, hypertension and ischemic heart diseases), drug abuse and history of hospitalization in ICI with NIHSS rate (P > 0.05). 106 patients (75.71%) have never taken any type of statin class of drugs and only 34 patients (24.28%) were taking these medications before the stroke. The difference between NIHSS rates of groups A and B was not clear and notable (P > 0.05).

**Conclusion.** According to the findings of this research, taking statins before the onset of stroke and immediately after it showed no significant difference in the level of following dysfunction, measured with NIHSS, and further studies are necessary.

## Introduction

Cerebrovascular attack (CVA) is an example of the main leading reasons of inability among adults in the United States. About 0.4% of the people in the United States, Australia, and Europe, over 45 years old, have their primary stroke at each 12 months [**1**]. After cardiovascular events, stroke is the secondary most frequent reason for the loss and is a principal vital reason for taken inability. In some communities, the mixed frequency of transient ischemic seizures and stroke passes the cardio-vascular events incidence [**2**]. Although many advances in defensive policies and primary treatment for stroke are occurring, approximately 750,000 strokes happen each year in America [**1**,**3**]. The risk of stroke is larger between whites and blacks, more frequent in men than in women, and is more prominent than in younger teams. More than 85% of the mortal strokes happen in low- and middle- business regions [**4**]. Stroke is considered in one of every 18 losses in America. The price of the detailed application is between the fastest-growing prices for Medicare. In 2007, approximately 25 billion dollars had been spent for medical care in the United States [**1**].

A report showed that the 30-days mortality after ischemic stroke for people 45 to 64 years old is of 8 to 12%. The likelihood of improvement and gaining previous physical activity quality after stroke changes with the quality and rigor of the original debt. Almost 35% of the heirs with the original disease of the leg do not recover a valuable function, and 20-25% of all the heirs are incapable of walking without substantial aid [**2**]. In half-year after stroke, which is prevalent in more than 65% of the cases, patients cannot include the changed hand to their normal actions. Poor upper-extremity results are anticipated after a hemispheric infarction when the hand has no movement and the lower-extremity cannot move for two weeks or just slight finger flexion, compatible with the significant loss of the corticospinal field [**5**]. In the heart research of Framingham, between the survivors of an ischemic stroke, half of them had some sign of hemiparesis, 30% were incapable of walking without help, 19% had aphasia, and 26% were ordered [**1**].

Cases with stroke are at a potential risk of different vascular phenomena, like intermittent stroke (highest risk), cardiovascular events, and death from vascular causes. The conclusions of a meta-investigation discovered that the risk of stroke was as high as 12.8% over the initial seven days after transitory ischemic strokes, though the risk would be decreased when trained stroke services provided emergency medicine. It was calculated that at least 80% of the recurrent events might be restricted to the application of a wide method that comprised dietary adjustment, blood-pressure-lowering, training, antiplatelet treatment, and statin treatment [**6**]. Statin therapy is a new way of producing neuroprotective effects after acute cerebral ischemia. Statins (Hydroxymethylglutaryl-CoA reductase inhibitors) are useful in the initial and subsequent inhibition of stroke and acute coronary issues. Besides diminishing the level of lipids, statins have other important actions, including anti-inflammatory, vasodilatory action, antithrombotic, and antioxidant effects [**4**]. Some studies showed that statin treatment at stroke onset and the novel started statins are linked with the modified before and late findings. But, some other studies found that there is no relation between the use of statins and improved outcomes [**6**,**7**]. Functional scales, such as the National Institutes of Health Stroke Scale (NIHSS) tend to accurately show and quantify the impairment produced via a stroke. The NIHSS has been found to be an excellent tool in predicting the patient’s results. A NIHSS rate of more than 16 shows a substantial possibility of dying, whereas a NIHSS rate lower than 6 shows a substantial possibility of a real improvement. On the average, an addition of 1 score in the NIHSS rate reduces the possibility of a real result by 17 percent [**6**].

No similar domestic study about statins usage and their effects was found. Also, because of the controversies in the impact of statins on the results of the cases with stroke and not enough studies on the association between statins and NIHSS in patients, it was decided that the impact of statin use on NIHSS in CVA patients in Bandar-Abbas would be evaluated in this research.

## Material and Methods

140 patients with ischemic stroke were evaluated in this descriptive research, which was undergone in 2014, in Shahid Mohamadi Hospital in Bandar Abbas-Iran. The target group was made up of all the patients who were diagnosed with an ischemic stroke and, based on the World Health Organization’s (WHO) description of ischemic stroke, patients with first and multiple episodes of stroke were recruited to take part in this study. Participants were selected via convenience sampling. Before performing any examination or asking questions, the aim and method of the study were described to the patients. Their agreement was necessary to allow their participation in the study. The exclusion criteria were the following: non-neurologic blindness, intracranial hemorrhage and no tendency to take part in the study. Primary examination and workups were conducted in the first hours after the admission in the emergency room. The neurologist examined patients with primary diagnosis of transient ischemic attack (TIA), cerebrovascular accident (CVA), and stroke. Required data were collected after the confirmation of the ischemic stroke. Three types of information were collected; 1- demographic (age, gender), 2- medical (previous and underling disease, drug history, lab data and smoking or opium addiction), 3- stroke severity. NIHSS was employed to measure the stroke severity. This score was measured as soon as possible after the diagnosis of ischemic stroke. NIHSS is a functional test that qualifies the impairment caused by stroke. NIHSS has 11 parts: 1- Consciousness stage (LOC) (contains: LOC responsiveness, LOC surveys and LOC orders), 2- Horizontal eye action, 3- Visual area exam, 4- Motor arm, 5- Facial palsy, 6- Limb ataxia, 7- Motor leg, 8- Language, 9- Sensory, 10- Extinction, and 11- Speech. Each part evaluates a skill of the examinee and has a 0-4 score. The final points are created from the addition of every part’s score. The minimum point is 0 and the maximum is 42, a higher score showing a higher degree of the impairment. Score 0 means no stroke symptoms, 1-4, 5-15, 16-20, and 21-42 means minor stroke, moderate stroke, and severe stroke. After collecting the information, all the data are entered into SPSS version 23. Because our data had a non-normal distribution, descriptive, Mann Whitney and Spearman correlation tests were conducted. 

## Results

The objective of this research was to evaluate the statin effects on NIHSS in 140 cases with ischemic stroke, referred to Shahid Mohammadi Hospital of Bandar Abbas in 2014. The patient information, which was collected by using a standard questionnaire to measure NIHSS and the researcher’s checklist to record the clinical and demographic data, were inserted into SPSS Statistical Software Version 23 and the essential statistical analysis was made. At first, the NIHSS variable was evaluated by using the Kolmogorov-Smirnov test to see whether the distribution was normal or not. It was found that the distribution was abnormal (sig = 0.000). Totally, 95 men and 45 women, with the average age of 68.5 and 66.7 years old, were studied. There was no clear distinction between the two groups (women and men) regarding age (P > 0.05).

The correlation between the age and NIHSS was studied by using the Spearman Test and a direct correlation was noticed between these two variables (sig = 0.075). However, it was not significant (P > 0.05). There was no clear link among the sex and the NIHSS rate (P > 0.05). 

The number of strokes was studied between the participants (**Table 1**). 

**Table 1:** Episodes of stroke incidence

	Episodes of stroke incidence	Frequency
No previous incidence	83	59.3%
1 time	41	29.3%
2 times	12	8.6%
3 times	1	0.7%
4 times	3	2.1%

According to the studies, there was no significant relationship between the type of stroke and the NIHSS rate (P > 0.05) while a reverse but insignificant relationship was noticed between the number of stroke incidences and the NIHSS rate (sig = 0.598, P > 0.05). 

Other parameters studied in these patients included the serum levels of Hb, BS, cholesterol, TG, LDL, HDL, and Plt. A reverse relationship was achieved between these parameters and the NIHSS rate, which was not statistically significant (P > 0.05). There was only a direct correlation between the serum HDL level and the NIHSS rate which was also not significant (P > 0.05) (**Table 2**).

**Table 2:** Laboratory data

	HDL	LDL	Chol	TG	BS	Hb	PLT
NIHSS Correlation Coefficient	0.108	-0.043	-0.019	-0.104	-0.037	-0.015	-0.133
Sig	0.430	0.751	0.890	0.447	0.671	0.861	0.124

**Fig. 1:**
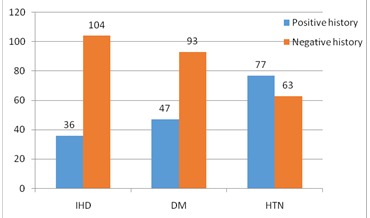
Underlying disease

Underlying diseases, including ischemic heart diseases, diabetes, and hypertension were also studied in patients. The frequencies of the mentioned diseases were illustrated in **Fig. 1**. There was no significant relationship between the underlying diseases and the NIHSS rate (P > 0.05).

Finally, the NIHSS rate was studied in two groups of patients with the history of taking statins and those with no history of taking statins before the stroke. 106 patients (75.71%) have never taken any statin medications while only 34 (24.28%) have taken them before the onset of stroke. There was no significant relationship between the status of taking statins before and after the onset of stroke and the NIHSS rate (P > 0.05). 

## Discussion and conclusion

Based on the previous studies, statins are effective in lowering the probability of stroke incidence among those with the history of stroke and those with no history of stroke but with cardiovascular risk factors. In addition, statins could improve the outcome of these patients in a way that could decrease the brain infarct volume in experimental studies [**8**,**9**]. Taking statins before the onset of strokes could decrease the neurologic disorders after a localized infarction in the brain. In mice, which were treated with statin 24 hours after focal brain ischemia, the neurological activity, synaptogenesis, angiogenesis, and the migration of neural precursor cells in infarcted areas, were more prevalent than in the control group [**10**]. Other experimental studies indicated that statins could act as vasodilators, have antithrombotic and anti-inflammatory effects, and protect the nervous system. These could be taken into account to promote the clinical conditions of patients suffering from strokes [**10**,**11**]. In Chroiniet et al. study, the early treatment with statin, survival and functional outcomes of patients with ischemic strokes were studied. The survival was significantly increased during the first 7 days after the stroke in the group with patients who experienced the stroke for the first time, while treating statins 72 hours after its onset. Taking statins routinely before the stroke during the last year was considered in the study and there was also no significant relationship between the prior taking of statins and the survival during the first 7 days after the stroke. However, aspirin was also used as an additional treatment in the study. Aspirin effects alone and/ or with statins is also a controversy [**12**]. Other parameters considered in this study as effective on the patient’s survival were age, blood pressure, NIHSS, and the functional level before the stroke. Of them, aging, hypertension, high NIHSS rate and low physical activity before the stroke incidence respectively decreased the survival [**12**].

On the other hand, Moonis et al. divided the patients into three groups to study the impacts of statins on promoting the clinical outcomes of those suffering from ischemic stroke. The first group took statins before the onset of stroke, the second group began to take statins after the stroke, and the third group has never taken statins. The primary NIHSS averages in groups were 13.1 ± 3.8, 12.9 ± 4.1 and 13.1 ± 3.7, respectively. There was no clear distinction [**13**]. The clinical outcomes of the groups were studied and finally, the group that began to take statins after the stroke showed a better clinical outcome (P > 0.007). Diabetes, prior episode of stroke or TIA and taking anti-hypertensive drugs were the parameters that caused a poor clinical outcome. On the whole, statins showed a positive effect on the patient’s clinical outcome based on the results of this study [**13**]. However, the NIHSS rate was measured upon admitting the patients hospitalized for stroke diagnosis in our study, and, like in the study of Moonis et al., no significant relationship was found between the 2 groups taking statins earlier and before the stroke (P > 0.05). The reasons for the above-mentioned difference in other studies and our study, between the groups taking statins before and after the stroke might be the following: different races, drug dosage, potency of medication therapy and/ or the duration of taking statins before the onset of stroke. Some parameters that might affect the patient’s clinical outcomes including age, sex, history of stroke, underlying diseases (diabetes, hypertension and ischemic heart diseases), drug abuse, lipid profile (TG, HDL, LDL, Total Cholesterol) were also studied, and none showed significant relationships with the NIHSS rate (P > 0.05). Meanwhile, parameters such as age, diabetes, and hypertension have affected the patient’s clinical outcomes in the previous studies. The reason beyond this difference might be the long-term follow up of the cases in other studies, since the patients were only examined once in our study.

## Conclusion

Based on the results of the study, no significant relationship was seen between taking statins before the stroke and the beginning of taking statins immediately after the stroke, with NIHSS (P > 0.05). Meanwhile, there was no clear relationship among age, sex, history of stroke, lipid profile (TG, HDL, LDL, Total Cholesterol) and the underlying diseases (diabetes, hypertension and ischemic heart diseases) with NIHSS rate in the two groups (P > 0.05).
